# Population Structure and Genetic Diversity Among Shagya Arabian Horse Genealogical Lineages in Bulgaria Based on Microsatellite Genotyping

**DOI:** 10.3390/vetsci12080776

**Published:** 2025-08-19

**Authors:** Georgi Yordanov, Teodor Yordanov, Ivan Mehandjyiski, Georgi Radoslavov, Delka Salkova, Peter Hristov

**Affiliations:** 1 Department “Special Branches”, Institute of Animal Sciences, Agricultural Academy, 2232 Kostinbrod, Bulgaria; g.yordanov@iasrj.eu; 2 Bulgarian Horse Breeding Society, 1756 Sofia, Bulgaria; t_akb@abv.bg; 3 Research Center of Stockbreeding and Agriculture, Agricultural Academy, 4700 Smolyan, Bulgaria; bg_porodi07@abv.bg; 4 Department of Animal Diversity and Resources, Institute of Biodiversity and Ecosystem Research, Bulgarian Academy of Sciences, 1113 Sofia, Bulgaria; gradoslavov@gmail.com; 5 Department of Experimental Parasitology, Institute of Experimental Morphology, Pathology and Anthropology with Museum, Bulgarian Academy of Sciences, 1113 Sofia, Bulgaria; dsalkova@abv.bg

**Keywords:** Shagya Arabian horse, genetic differentiation, population genetics, microsatellites

## Abstract

The Shagya Arabian horse breed was created to address the need of Imperial Hussars (Hungarian light horsemen) for a horse with the intelligence, essential characteristics, and endurance of the Arabian breed, but also of a bigger size and having a better weight-carrying capacity and jumping ability. The present study aimed to explore the genetic variability and population structure of the uninvestigated Shagya Arabian horse population in Bulgaria based on genotyping at 15 equine microsatellite markers. A total of 140 horses belonging to six genealogical lines (Dahoman, Gazal, Ibrahim, Kuhailan Zaid, O’Bajan, and Shagya) were included in the survey. Genetic distances, analysis of molecular variance, principal coordinates analysis, and a Bayesian method were applied. The mean number of alleles in the individual subpopulations ranged from 3.67 in the Shagya to 5.13 in the Ibrahim sire line. The F_IS_ index was negative or close to 0 for the entire population and was −0.202. The overall *F*_ST_ was 0.014, indicating a low level of genetic differentiation between the subpopulations. The results of the principal components and the STRUCTURE analysis showed some level of admixture among the subpopulations in almost all genealogical lines. However, structural analysis also indicated a genetic similarity between the Ibrahim, Kuhailan Zaid, and Shagya lineages, while it showed a completely different genetic profile regarding the other three sire lines. Due to the higher admixture and the discovery of more distinct genetic clusters, it can be assumed that there is a higher gene flow from one lineage to another in the Shagya Arabian horse population in Bulgaria and that there is sufficient genetic variability and diversity to suggest adequate measures for preserving this rare breed. In addition, this study may highlight the risk of the loss of gene diversity in this population and help to implement suitable breeding programs to preserve genetic diversity.

## Simple Summary

Microsatellites are among the most frequently used genetic markers for assessing genetic diversity and structure within and between populations. Their high polymorphism, codominant nature, and relatively easy amplification through PCR make them popular for these studies. In the present study, we evaluate the genetic structure and differentiation of six sire lineages belonging to the Shagya Arabian horse breed through microsatellite genotyping. We found significant genetic diversity, but low genetic differentiation and a high level of admixture among sire lines. These findings allowed the creation of a breeding program for the protection and preservation of this horse. The loss of a breed represents not only the disappearance of a genetic heritage but also the loss of an important piece of equestrian culture.

## 1. Introduction

In the late 1700s, the Austro-Hungarian military perceived the need to improve the quality of its mounts; hence, officials set out to breed the ideal cavalry horse [1]. Their aim was to preserve the soundness, hardiness, stamina, and elegance of Arabian horses while producing a larger horse with more bone, powerful hindquarters, a more rounded topline, and a calm and workmanlike disposition. In 1789, a desert-bred Arabian stallion named Shagya was imported from Syria to Babolna, the Hungarian state stud [2]. Shagya Arabians, as the resulting breed was called, have been bred there up to the present time. Shagya’s sons were crossed with local mares of Thoroughbred, Lipizzaner, and oriental ancestry. Predominantly gray in color, Shagya Arabians can also be bay, chestnut, or black. Shagyas should be beautiful and harmonious, with expressive heads, well-formed necks, good toplines, long croups, well-carried tails, and strong, correct legs. The breed was officially accepted and approved by WAHO in 1978. The Internationale Shagya-Araber Gesellschaft was formed in 1983 as the governing body for the breed [3]. Today’s breeding goal is still a beautiful Arabian horse with a noble appearance, which can be used for riding and driving, while also meeting the temperament and performance requirements of leisure and equestrian sports, endurance, and hunting.

In Bulgaria, the beginning of the development of the Shagya Arabian breed dates back to 1894 with the opening of the Stud farm “Kabiuk” and the establishment of the Arabian section [4]. The designation of the stud was to create and select a native military horse for the needs of the army and to provide breeding material for improving countryside horse breeding. Initially, a few stallions and mares were bought from the most prestigious studs in Poland. Afterward, four other half-purebred Arabian mares as well as a stallion (considered as one of the founders of the Shagya type) were imported from the Radautz stud [5]. In 1906, 12 mares and 3 stallions were imported from the Strelets stud (Luhansk district, Ukraine). In 1926, two stallions, representatives of the original Arabian line, Siglavi Bagdady, were bought from Hungary [5]. At the beginning of the 1930s, a selection was directed towards increasing the height of Shagya Arabians. For this purpose, in 1933, two half-thoroughbred stallions, descendants of the Shagya stallion, were bought from the Babolna stud. During WWII, the size of the breeding Shagya herd was reduced. In 1949, two thoroughbred Arabian stallions and one of the Shagya type were imported from Poland. After WWII, the need for riding horses diminished, and agricultural mechanization led to a reduction in the Shagya Arabian herd [5]. In the early 1970s, priorities were directed towards the improvement of the exterior of horses, which resulted in the regular import of stallions and mares from the Arabian horse breed. At the beginning of the 1980s, the price of Arabian horses considerably increased, and the idea of the gradual replacement of half-thoroughbred mares by purebred mares came forth. In 1989, the use of Shagya stallions started again, and nowadays, the higher percentage of blood from Arabian ancestors has contributed to the consolidation of a very well-expressed eastern typicality of Shagya horses in Bulgaria.

Some of the most commonly used genetic markers for assessing genetic diversity and population structure in different taxa of organisms are microsatellite markers [6,7,8,9]. In addition, they also provide information regarding domestication processes [10], population differentiation [11], genetic bottlenecks and founder effects [12], hybridization studies [13], assessment of genetic rescue [14], etc. Single nucleotide polymorphisms (SNPs) based on traditional DNA sequencing [15] have long been established, but unlike microsatellites, they have been used relatively seldom in population genetics. Also, microsatellite mutation rates are several orders of magnitude higher and much more variable than those of SNPs [16]. Furthermore, their (mostly) bi-allelic state limits the information content per locus in comparison with the more polymorphic microsatellite markers [17].

The genetic structure and diversity of many horse breeds are important for maintaining and pursuing breeding goals for genetic improvement [18,19,20]. In this context, microsatellites are often used to evaluate the genetic diversity, population structure, and conservation status of a large number of different horse breeds [21,22,23,24,25,26]. The stability, ease, and accuracy of genotyping these codominant markers, as well as their wide distribution in the genome, make microsatellite loci a valuable source of information regarding genetic diversity and population structure [27,28,29].

The Shagya Arabian horse is found worldwide, but breeders are still primarily concentrated in Europe, including Hungary, Romania, Poland, Czechia, Germany, Switzerland, Austria, Bulgaria, etc. (https://www.fei.org/stories/lifestyle/my-equestrian-life/breed-profile-shagya-arabian, accessed on 12 August 2025). Nevertheless, only two scientific papers focused on the evaluation of the genetic structure and diversity of the Shagya Arabian horse have been published so far [30,31]. The scarce genetic research on this famous breed motivated us to conduct the present study. Therefore, the aims of this study were to investigate the genetic diversity of the Shagya Arabian sire lines available in Bulgaria, to analyze the diversity within and among subpopulations, and to evaluate the genetic structure of each of them using microsatellite markers.

## 2. Materials and Methods

### 2.1. Animal Welfare and Ethical Statement

All experimental procedures were reviewed and approved by the Animal Research Ethics Committee of the Bulgarian Food Safety Agency (BFSA) (Art. 154 of the Law on Veterinary Activity), in accordance with European Union Directive 86/609.

### 2.2. Sample Collection

**Figure 1:**
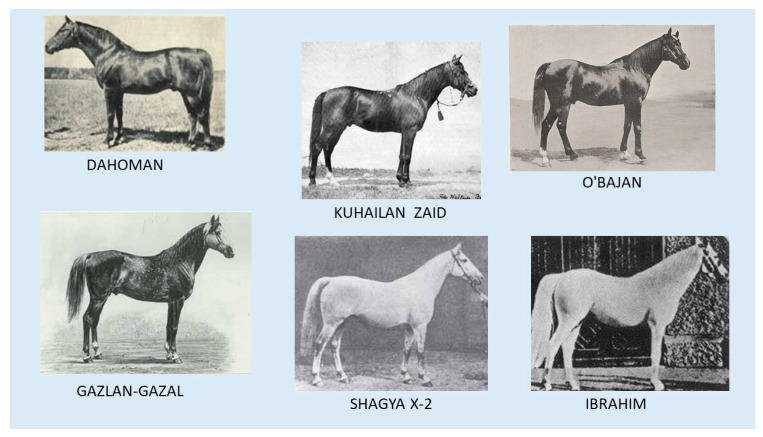
Pictures showing the six Shagya Arabian founders investigated in this study.

A total of 140 hair samples were obtained from the manes and/or tails of Shagya Arabian horses (male and female) in 2020–2025 for parentage verification control. The samples were preserved under dark storage in filter paper packages, with silica gel to dehydrate the samples in order to prevent degradation. The samples represented six Shagya Arabian sire lines, including Dahoman (DAH, *n* = 25), Gazal (GAZ, *n* = 24), Ibrahim (IBR, *n* = 37), Kuhailan Zaid (KUH ZAID, *n* = 21), O`Bajan (O’BAJ, *n* = 25), and Shagya (SHA, *n* = 8) (Figure 1). The selection of samples was designed according to two main factors. First, the chosen farms should have a reputation and credibility in terms of breeding of the sire line subpopulation available. Thus, our DAH, KUH ZAID, O’BAJ, and SHA samples originated mainly from the “Kabiuk” Stud farm (Shumen, Bulgaria). The GAZ and IBR samples were taken either from that stud or from private breeders. Second, we selected horses not related to each other for at least three generations, with phenotypical traits of the Shagya Arabian horse breed, all registered in the WAHO stud book. All information and pedigree data were verified by the Bulgarian national stud book, available in the Executive Agency for Selection and Reproduction in Animal Husbandry (Sofia, Bulgaria). All samples with a doubtful or excluded pedigree were eliminated.

### 2.3. DNA Extraction and Fragment Analysis

Genomic DNA was isolated from the hair follicles of about 20 hairs, using a Tissue DNA purification kit (Cat. No. E3550, EURx Ltd., Gdansk, Poland), according to the manufacturer’s instructions. The quantity and quality of the extracted DNA were evaluated spectrophotometrically and on 1% agarose gel electrophoresis staining with SimpliSafe™ (Cat. No. E4600; EURx Ltd., Gdansk, Poland) under UV light. After that, the DNA was stored at −20 °C prior to analysis.

A total of 15 microsatellite markers (AHT4, AHT5, ASB2, ASB17, ASB23, HMS1, HMS2, HMS3, HMS6, HMS7, HTG4, HTG6, HTG7, HTG10, and VHL20) specific to *Equus caballus* were used in the present study (Table 1). All markers are included in the panel recommended by the International Society for Animal Genetics (ISAG) and the Food and Agriculture Organization of the United Nations (FAO UN) for molecular genetics diversity studies and parentage testing.

All samples were sent to the GeneControl GmbH laboratory (Grub, Germany), where PCR amplification and fragment analysis were performed. The 15 microsatellites were amplified in one multiplex reaction. The primers used for multiplexed PCR were labeled using FAM, VIC, and NED standards. The fragment sizes of the microsatellite alleles were determined using an ABI3130XL genetic analyzer (Applied Biosystem, New York, NY, USA). A Gene Scan 500 ROX-labeled size standard was used for internal size standardization of the obtained PCR products. The data obtained were further analyzed using GeneMapper 5.0 software (Applied Biosystem, New York, NY, USA).

### 2.4. Statistical Analyses

#### 2.4.1. Genetic Diversity Within and Among Sire Lineages

The standard parameters of genetic diversity, including the average number of identified alleles per locus (Na), the average number of effective alleles per locus (Ne), the observed (Ho) and expected (He) heterozygosities, the Hardy–Weinberg equilibrium across loci, the Shannon’s information index (I), the fixation index (F), and the F (Null)–Null allele frequency, were calculated using GenAlEx 6.5 (New Brunswick, NJ, USA) [38]. GenAlEx 6.5 was also used to perform the principal coordinate analysis (PCoA) based on the genetic distance matrix, calculated by the Nei genetic distance within the entire population as well as among all six Shagya Arabian horse lineages [39]. Allelic richness (AR), which could serve as an alternative criterion for measuring genetic diversity [40], was estimated by using an HP-Rare program [41]. Wright’s *F*-statistics, according to [42], were applied to calculate the *F*_ST_, *F*_IT,_ and *F*_IS_ parameters for each locus and each Shagya Arabian sire line. The polymorphic information content (PIC) of each locus was calculated using the equation of Botstein et al. [43], utilizing Cervus v3.0.7 software [44]. Finally, a variance analysis (AMOVA) was performed for different partitions of the eight Shagya Arabian horse subpopulations, using GenAlEx 6.5, where the variation among subpopulations was determined by the interpopulation genetic distances based on 999 permutations.

#### 2.4.2. Population Structure and Individuals Assignment

STRUCTURE 2.3.3 software [45] was used to explore the relationships among the six Shagya Arabian lineages and to assign the samples into clusters, applying the Bayesian method under an admixture model. Different values for the length of the burn-in period (20,000–50,000) and Markov chain Monte Carlo (MCMC) repetitions (100,000–150,000) were tested. Different K values between K = 2 and K = 12, where K is the number of tested clusters, were applied. Runs for each K were repeated 10 times. The software Clumpack was used to align multiple replicates for each K in order to facilitate the interpretation of the clustering results [46]. The DISTRUCT application [47] was used to display the results graphically. The best number of clusters was determined depending on the ΔK value [48], which was calculated and plotted using the StructureSelector application [49].

## 3. Results

### 3.1. Polymorphism of Microsatellite Markers

All 15 loci used in the present study were found to be polymorphic in the entire Shagya Arabian population (Table 2). Within the entire population of 140 individuals, a total of 409 different alleles were detected, with a mean number of alleles per locus of 4.54 ± 0.15. The locus ASB2 showed the highest number of alleles (7.5), while the lowest number of alleles was found for the locus HTG7 (2.0), followed by HTG6 (3.0). The locus ASB2 (nine alleles) was the most polymorphic locus in the DAH subpopulation, whereas the loci HTG7, HTG6, HMS6, and AHT5 showed low polymorphism (Supplementary Table S1). Interestingly, the locus HTG7 showed the lowest polymorphism, with two alleles, in almost all sire lines except the SHA lineages, where it was represented by one allele (monomorphic). This result is most likely due to the small sample size of the indicated line. The mean effective number of alleles of the entire horse population was 2.98, varying from 2.76 (SHA line) to 3.13 (O’BAJ lineages) (Table 3). Excluding the HTG7 locus in the SHA line, which was monomorphic, the HTG marker also showed a very low Ne in all the studied lines, ranging from 1.22 (DAH lineage) to 1.92 (GAZ line) (Supplementary Table S1). The other loci with low Ne values were HMS2, with 1.29 and 1.42 in KUH ZAID and SHA, respectively, and ASB23 and AHT5, both with 1.78 in the GAZ and DAH lineages. The private alleles were only found in the DAH line for the locus ASB2 (1) and four alleles in the IBR lineages (HMS2, AHT5, HTG7, and HMS1 loci). The polymorphic information content (PIC) varied from 0.30 for the locus HTG7 to 0.84 for the marker VHL20, with an average value of 0.63 (Table 2). The only locus showing a value of less than 0.5 was HTG7, suggesting that it is not a suitable marker for assessing the genetic diversity of the Shagya Arabian horse in Bulgaria. The estimated null allele frequency (F Null) slightly varied from negative to positive values, with an average close to zero (−0.0098), assuming only a very low genotyping error rate. The allelic richness (AR) is a measure of genetic diversity, which provides information about a population’s long-term potential for adaptability and persistence. The lowest value of AR was 3.63, which was found in the SHA subpopulation, while the highest value was found in the O’BAJ sire line (4.01) (Table 3).

### 3.2. Levels of Heterozygosity and F-Statistics

The calculated average He and Ho values were 0.62 and 0.69, respectively; the He values varied from 0.27 (HTG7) to 0.80 (ASB2), while the Ho values ranged from 0.29 (HTG7) to 0.87 (HTG10) (Table 2). Also, the average He in the Shagya Arabian horses ranged from 0.57 (SHA) to 0.65 (O’BAJ). The Ho values showed the highest value in the SHA lines (0.71) and the lowest in the KUH ZAID lineage (0.65) (Table 3). The distribution of H_O_ and He among the sire lines was not significantly different between the subpopulations (Kruskal–Wallis chi-squared = 4.86, df = 5, *p*-value = 0.43, *p* ≥ 0.05; chi-squared = 5.0, df = 5, *p*-value = 0.41, *p* ≥ 0.05, respectively). A similar insignificant difference was found regarding the Na value among the Shagya Arabian sire lineages (chi-squared = 5.0, df = 5, *p*-value = 0.41, *p* ≥ 0.05).

The values of the fixation index (*F*_ST_) for individual loci ranged from 0.73 (HMS2) to 0.124 (ASB23), with an average value for the entire population of 0.077 (Table 4). We assume this value to be close to zero. This suggests that very little genetic differentiation exists within the Shagya Arabian subpopulation, whereas high genetic similarity among the six sire lines was observed instead. The global deficit of heterozygotes (*F*_IT_) across the entire Shagya Arabian population was −0.027. The *F*_IT_ in our case was below zero, indicating excess heterozygosity. Also, this value denotes only around 3.0% more observed homozygotes across all the subpopulations. The values of the inbreeding coefficient (*F*_IS_) were very low and negative (−0.213 HTG10 to −0.048 HMS7). The average value of *F*_IS_ in the 15 analyzed loci was 8 × 103, indicating a low level of inbreeding in the studied population of the six Shagya Arabian sire lines (Table 4). A negative *F*_IS_ value suggests that there are more heterozygotes (individuals with two different alleles for a gene) than would be expected if the population were in Hardy–Weinberg equilibrium and/or in cases of small sample sizes of some sire lineages or non-random sampling.

### 3.3. Hardy–Weinberg Equilibrium (HWE)

Microsatellite loci were tested for deviations from HWE, and the results are presented in Supplementary Table S2. Only two out of the fifteen studied microsatellites showed deviation from the Hardy–Weinberg Equilibrium test in the whole population. Deviation from HWE was observed only in the KUH ZAID sire line for loci ASB2 (*p* < 0.05) and HMS2 (*p* < 0.05). In the remaining loci, no deviation from HWE was observed. These results indicate that uncontrolled mating in the history of almost all lineages has not occurred.

### 3.4. Genetic Differentiation and Distance Among Shagya Arabian Sire Lines

The *F*_ST_ value was calculated in order to analyze the degree of differentiation among the studied Shagya Arabian subpopulations (Supplementary Table S3). The highest *F*_ST_ was detected between DAH and GAZ (0.065), followed by GAZ and SHA (0.060), DAH and SHA (0.057), etc. The lowest *F*_ST_ was observed between IBR and KUH ZAID (0.028), DAH and IBR (0.030), and IBR and O’BAJ (0.036).

The obtained values of genetic distance are shown in Supplementary Table S3. The highest values of genetic distance were observed between DAH and GAZ (0.259), DAH and O’BAJ (0.234), and GAZ and KUH ZAID (0.202). These values correspond to the differences in the studied microsatellite loci at the genome level, expressed as differences in the allele lengths and, respectively, their frequencies. The lowest values were detected between IBR and KUH ZAID (0.097), DAH and IBR (0.111), and IBR and O’BAJ (0.148).

We also conducted an analysis of molecular variance (AMOVA) to determine how much of the total genetic variation in a species is distributed between different levels of the population structure (Supplemental Table S4). As expected, most genetic variation (59%) was retained within the individuals in each sire line, whereas only 2% (*p* < 0.01) of the variation was among the studied subpopulations. The coefficient of genetic differentiation calculated based on this analysis (*F*_ST_ = 0.077) is an indication of a low level of differentiation among the sire lines, which may serve as a starting point for further monitoring of the dynamics of each Shagya Arabian lineage for future development and the process of their differentiation at the genetic level.

### 3.5. Genetic Structure and Genetic Relationships Among Shagya Arabian Sire Lines

**Figure 2:**
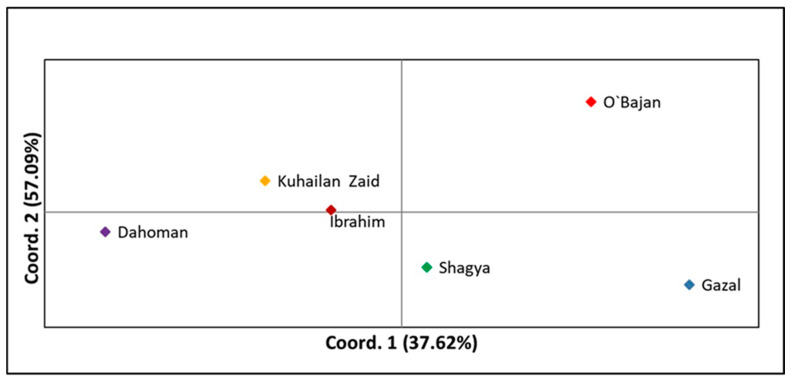
Principal coordinate analysis (PCoA) of the six Shagya Arabian horse lineages implemented in GenAlEx across the two principal components (PCoA1, PCoA2). Abbreviations are listed in Table 3.

**Figure 3:**
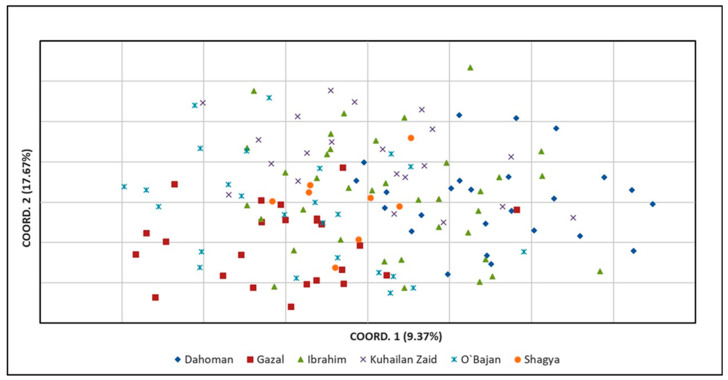
Principal coordinate analysis (PCoA) of the entire Shagya Arabian horse population, created by GenAlEx across the two principal components (PCoA1, PCoA2). The two-dimensional plot of the PCoA analysis illustrates the clustering of the 140 Shagya Arabians. Abbreviations are listed in Table 3.

Principal coordinate analysis (PCoA) was applied to analyze the genetic relationships among the tested sire subpopulations as well as within the entire Shagya Arabian horse population. As shown in Figure 2, the highest genetic relatedness was observed between the KUH ZAID and IBR sire lines. The SHA and DAH lineages clustered near them but were genetically distinct compared to KUH ZAID and IBR. Otherwise, O’BAJ and GAZ clearly differentiated from the other sire lines. These results could be expected, considering the levels of genetic differentiation between the individual lines presented in Supplementary Table S3. When comparing the entire Shagya Arabian population, some differences were observed (Figure 3). Many individuals of a given line were separated from the other lineages, but overall, genetic admixture was observed, most likely due to the low genetic differentiation (0.077).

**Figure 4:**
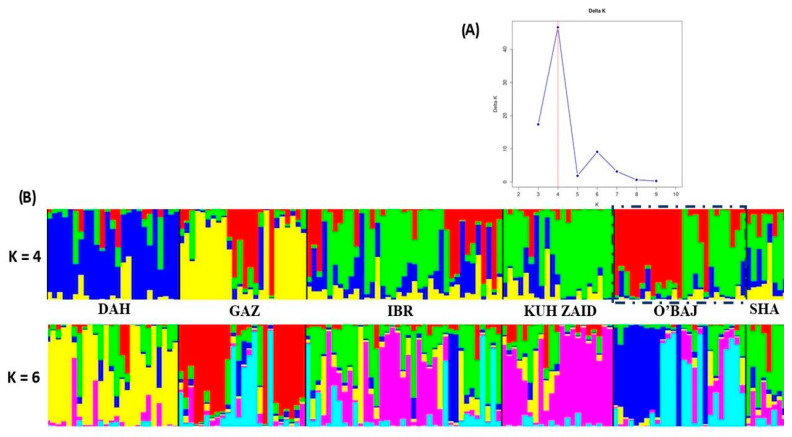
(**A**) Estimation of the most probable K using the delta K method by Evanno et al. [48]. The highest ΔK was found at K = 4. (**B**) Clustering assignment depending on the Bayesian method under an admixture model obtained by STRUCTURE software, using K = 4 and 6. Each individual is represented by a single column that is divided into segments whose size and color correspond to the relative proportion of the animal genome corresponding to a particular cluster. Populations are separated by black lines. The O’BAJ sire line (two subpopulations shown) is shown in a dashed box. The blue color included mainly the representatives of DAH sire line. The yellow individuals of GAZ lineage. The IBRq KUH ZAID, SHA and almost the half of O,BAJ sire lines represented by green color. The last half of the animals of O,BAJ lineages were visualized in red. Shagya Arabian horse lineage abbreviations are given in Table 1.

The genetic structure of the entire population represented by 140 Shagya Arabian representatives of the six studied sire lines was evaluated using STRUCTURE v 2.3.4. (Figure 4). When all the data were analyzed together, the optimal number of distinct genetic populations was based on the mean LnP(K) = 2.029 and ∆K = 30.082. The most probable number of genetic clusters, as determined by the delta K method, was four (Figure 4A). Evanno’s delta K method [48] showed another lower peak at K = 6. According to [48], the occurrence of several ΔK modes is indicative of sub-structuring within each of the studied sire lines, and the heights of each ΔK mode describe the strength of the genetic structure at each K. The clusters obtained from STRUCTURE analyses at K = 3 and K = 8 are shown in Figure 4B. The mixed colors with proportional lengths represent the admixture level for the defined Shagya Arabian subpopulations at K between 4 and 6. At K = 4, the entire Shagya Arabian population showed four main clusters. Cluster 1 included the DAH sire line, cluster 2—most individuals from the GAZ lineage, cluster 3—IBR, KUH ZAID, SHA, and about half the representatives from the O’BAJ lineage, and cluster 4—the rest of the O’BAJ line. The third cluster showed the largest admixture, since many individuals from the DAH and GAZ sire lines were observed therein. Similarly, many representatives from the O’BAL line were presented in the GAZ lineage. An interesting finding of this STRUCTURE analysis is the presence of two subpopulations in the O’BAJ sire lines. One of them was homogeneous and formed a completely different cluster (almost uniform red color), and the other one consisted of individuals showing genetic similarity to the IBR, SHA, and KUH ZAID lineages.

## 4. Discussion

This work presents the first description of the genetic diversity and population structure of six Shagya Arabian lineages in Bulgaria. Although this breed is widespread in a large number of countries, the overall populations amount to a small number of animals, which is probably due to the preference for other horse breeds. Therefore, it is not surprising that molecular genetic research on this breed is quite scarce.

### 4.1. Genetic Diversity Within and Among Shagya Arabian Sire Lineages

The results represent the genetic diversity and population structure of the Shagya Arabian horse breed in Bulgaria as a whole, as well as an individual assessment of each sire line therein. Since there are only two similar studies on this breed in the available literature, we decided to compare the obtained results with the published data on the Arabian horse, the breed based on which the Shagya Arabian was created.

The observed heterozygosity for the entire Shagya Arabian population was 0.62 (Table 2). The studied sire lines showed a high level of genetic diversity, considering the highest heterozygosity value (0.65) in the O’BAJ line and the lowest (0.57) in the SHA line (Table 3). These values were similar to those reported for the Hungarian Shagya Arabian (0.69) using the same or similar loci [30]. Furthermore, an analogous value was detected for other Arabian populations like Turkish Arabian (0.67) [50]; Egyptian Arabian horses (0.69) [51]; Desert breed, Straight Egyptian, and Polish Arabian (0.67, 0.64, and 0.64, respectively) [25]; and Saudi, Syrian registered, and Iranian Arabian (0.68, 0.69, and 0.71, respectively) [30]. One of the powerful tools to support resolution that depends on heterozygosity in different populations is the average number of alleles per locus (Na) [52]. It has been reported as the most relevant index in conservation programs [53,54]. The average number of alleles per locus was 4.54 (±0.15) (Table 2). Na ranged from 3.67 in the SHA sire line to 5.13 in the IBR sire line (Table 3). One of the disadvantages of the Na value is that it is considerably influenced by the sample size [55,56]; therefore, we also measured AR. AR showed the same pattern among all the tested subpopulations as Na, which indicates that the sample sizes for those Shagya Arabian sire lines had no noticeable effect on Na. The effective number of alleles (Ne) is another significant indicator of intra-line genetic diversity. The Ne for the entire Shagya Arabian horse population was relatively low (2.98), ranging from 2.76 (SHA) to 3.13 (O’BAJ), thus suggesting that all the subpopulations had a low level of genetic variability. In general, the Ne values observed in most Arabian populations have shown low indices. For example, Ne in Western and Middle Eastern Arabian horse populations varied from 2.35 (Egyptian–Saudi mix) to 3.51 (Syrian Arabian) [30], 3,34 (Turkish Arabian) [50], 3.69 (Egyptian Arabian) [51], etc.

The relatively low values of the F-statistics (*F*_IT_, *F*_ST_, and *F*_IS_; [42]) for individual loci and overall values (−0.113, −0.027, and 0.077, respectively) were close to zero. The estimated *F*_IS_ by [39] had similar values (−0.202). These results suggest that there is no reduction in heterozygosity in the Shagya Arabian population due to non-random mating within the total population and in the subpopulations or due to random genetic drift. The genetic differentiation over the loci was low (*F*_ST_ = −0.116), ranging from −0.230 (SHA) to −0.078 (IBR) (Supplementary Table S1).

### 4.2. Genetic Relatedness and Genetic Differentiation Among Shagya Arabian Sire Lineages

Analysis of Molecular Variance (AMOVA) based on F-statistic calculation is among the most frequently applied methods for determining a population’s genetic structure [57]. The AMOVA result in the present study suggested that the main source of variation came from within the individuals in the population (Supplementary Table S4). This indicates that the majority of the observed genetic diversity is found when comparing individuals within the same subpopulation, rather than when comparing subpopulations to each other. The genetic drift, natural selection, and gene flow were among the main factors influencing the results of these observations [58,59].

The genetic differentiation over the loci was close to 0 (*F*_ST_ = −0.116). This suggests low genetic differentiation among the subpopulations, most likely due to considerable gene flow or mixing of populations by origin. These values also suggest that the subpopulations are not sufficiently differentiated and may have a common history and/or breeding practices [7,11]. Very low genetic differentiation was observed in other Arabian populations, e.g., Turkish Arabian (0.074) [50], Arabian population in Bulgaria (0.096) [60], Syrian Arabian (0.016) [30], Egyptian Arabian (0.033) [51], Polish Arabian (0.020) [61], etc.

We also performed a principal coordinate analysis (PCoA) as the first approach to analyze the population structure of the entire Shagya Arabian population (Figure 2) as well as of the sire lines (Figure 3). As a whole, the outcomes from the PCoA matched the results explained by both the genetic relatedness among all individuals and the traditional genetic distances among the studied subpopulations. For example, the GAZ line showed the highest relatedness with the IBR and the O’BAJ lineages (Supplementary Table S3). This genetic proximity was visualized in the lower left square of the PCoA plot. However, the obtained results confirmed that the Shagya Arabian population in Bulgaria was not sufficiently differentiated and that there was a high level of admixture between individuals. The PCoA, as shown in Figure 3, clearly differentiated the DAH, O’BAJ, and DAH sire lines. The IBR and KUH ZAID lineages were clustered close to each other. The SHA line showed a greater genetic proximity to the IBR and KUH ZAID lineages.

### 4.3. Population Structure and Shagya Arabian Sire Line Assignment

A genetic STRUCTURE analysis was used as a second approach to reveal the clustering of the individual sire lines (Figure 4B). The Bayesian clustering analysis at the optimal value of ∆K = 4 corroborated the close relationship and the admixed structure in the IBR, SHA, and KUH ZAID sire lines, which supported the results of both the PCoA and the pairwise *F*_ST_ test. This suggests that those three subpopulations have high levels of gene flow or share the same origin. The other three lines—DAH, GAZ, and O’BAJ—showed separated clusters and lower genetic admixture. Also, the STRUCTURE analysis demonstrated that the DAH lineage was the most homogeneous, probably as a result of the conservative breeding in this subpopulation, which descended from a limited number of founders. In general, both results (PCoA and STRUCTURE analysis) confirmed that all the studied Shagya Arabian sire lines were not sufficiently differentiated and that there was a high level of admixture among them.

The most interesting clustering was that of the O’BAL sire line. Almost half of the individuals formed a completely different cluster compared to the others, while the last half of the animals revealed a genetic similarity between the BR, SHA, and KUH ZAID sire lines. These observations were supported by the highest He and the lowest *F*_IS_ among all sire lineages. These results require additional investigations so as to confirm which of the two subpopulations is authentic.

It should be noted, however, that despite their usefulness in various applications, microsatellite markers have certain limitations, e.g., high mutation rates leading to homoplasy, potential for undetected variation in flanking regions, and challenges in modeling mutational processes. These may result in difficulties in data interpretation and affect the reliability of allele frequency estimations.

## 5. Conclusions

The present study examined the genetic structure and diversity in the Shagya Arabian population in Bulgaria, based on a panel of 15 microsatellite markers. Since genetic data for this breed are very scarce, we compared the main diversity indices with those of the closely related Arabian horse breed. The results of the genetic diversity analysis of the entire population almost matched the results for the individual Shagya Arabian sire lines. The observed heterozygosity values were high in all subpopulations, which was confirmed by the low and negative *F*_IS_ value. This is an indicator that sufficient genetic variability is present in all subpopulations. The obtained results from the PCoA and STRUCTURE analysis indicated a low level of genetic differentiation and a high degree of admixture among all compared subpopulations. Regarding the presence of two subpopulations in the O’BAJ sire line, the addition of supplementary samples, ensuring an adequate sample size and even representation of regional subpopulations, should clarify the actual genetic profile and subpopulation structure. Additionally, these results will contribute to the accumulation of new genetic data for this rarely studied breed as well as enhance the effort to improve the management of the Shagya Arabian and preserve the diversity found therein.

## Figures and Tables

**Table 1:** Horse STR information used in this study: Locus name, chromosomal location, repeat motif, reference, primer sequences, and the size ranges of the amplicons.

Locus	Chrom.	Motif	Primer Seq. 5′–3′	FOR Primer Label	Amplicon Length (bp)	Ref.
AHT4	24q14	(AC)nAT(AC)n	F: AACCGCCTGAGCAAGGAAGT R: CCCAGAGAGTTTACCCT	NED	144–164	[32]
AHT5	8	(GT)n	F: ACGGACACATCCCTGCCTGC R: GCAGGCTAAGGAGGCTCAGC	VIC	126–144	[32]
ASB2	15q21.3-q23	(GT)n	F: CCACTAAGTGTCGTTTCAGAAGG R: CACAACTGAGTTCTCTGATAGG	VIC	216–250	[33]
ASB17	2p14-p15	(AC)n	F: ACCATTCAGGATCTCCACCG R: GAGGGCGGTACCTTTGTACC	FAM	87–129	[33]
ASB23	3q22	(TG)n	F: GCAAGGATGAAGAGGGCAGC R: CTGGTGGGTTAGATGAGAAGTC	NED	175–211	[34]
HMS1	15	(TG)n	F: CATCACTCTTCATGTCTGCTTGG R: TTGACATAAATGCTTATCCTATGGC	FAM	170–186	[35]
HMS2	10	(CA)n(TC)2	F: CTTGCAGTCGAATGTGTATTAAATG R: ACGGTGGCAACTGCCAAGGAAG	VIC	222–248	[35]
HMS3	9	(TG)2(CA)2TC(CA)n (TG)2(CA)2TC(CA)nGA(CA)5	F: CCATCCTCACTTTTTCACTTTGTT R: CCAACTCTTTGTCACATAACAAGA	FAM	148–170	[35]
HMS6	4	(GT)n	F: GAAGCTGCCAGTATTCAACCATTG R: CTCCATCTTGTGAAGTGTAACTCA	VIC	151–169	[35]
HMS7	1q25	(AC)2(CA)n	F: TGTTGTTGAAACATACCTTGACTGT R: CAGGAAACTCATGTTGATACCATC	NED	165–185	[35]
HTG4	9	(TG)nAT(AG)5AAG(GA)5 ACAG(AGGG)3	F: CTATCTCAGTCTTGATTGCAGGAC R: CTCCCTCCCTCCCTCTGTTCTC	FAM	127–139	[36]
HTG6	15q26-q27	(TG)n	F: GTTCACTGAATGTCAAATTCTGCT R: CCTGCTTGGAGGCTGTGATAAGAT	FAM	84–102	[36]
HTG7	4	(GT)n	F: CCTGAAGCAGAACATCCCTCCTTG R: ATAAAGTGTCTGGGCAGAGCTGCT	NED	118–128	[37]
HTG10	21	(TG)n TATC(TG)n	F: TTTTTATTCTGATCTGTCACATTT R: CAATTCCCGCCCCACCCCCGGCA	VIC	95–115	[37]
VHL20	30	(TG)n	F: CAAGTCCTCTTACTTGAAGACTAG R: AACTCAGGGAGAATCTTCCTCAG	NED	87–105	[36]

**Table 2:** Average number of identified alleles (Na), average number of effective alleles (Ne), Polymorphic Information Content (PIC), heterozygosity: observed (Ho) and expected (He), Shannon’s information index (I), fixation index (*F*_ST_), and F (Null)–Null allele frequency estimated.

Locus	Na	Ne	PIC	Ho	He	I	*F*_ST_	F (Null)
AHT4	5.17	3.73	0.77	0.78	0.72	1.43	−0.084	0.0086
ASB2	7.50	5.09	0.82	0.86	0.80	1.77	−0.077	−0.0159
HMS2	5.00	2.18	0.54	0.51	0.49	0.99	−0.041	0.0237
HMS7	4.67	3.08	0.69	0.79	0.66	1.25	−0.184	−0.0354
HTG6	3.00	2.29	0.51	0.61	0.55	0.93	−0.102	0.0010
AHT5	3.67	2.30	0.54	0.64	0.55	0.95	−0.157	−0.0396
ASB23	4.50	2.82	0.65	0.63	0.59	1.13	−0.078	0.0352
HMS3	5.33	2.81	0.68	0.74	0.64	1.27	−0.156	−0.0199
HTG10	4.83	3.57	0.74	0.87	0.71	1.38	−0.222	−0.0473
HTG7	2.00	1.45	0.30	0.29	0.27	0.44	−0.081	0.0091
ASB17	5.00	3.18	0.67	0.76	0.68	1.32	−0.112	−0.0287
HMS1	4.17	2.73	0.59	0.71	0.62	1.11	−0.133	−0.0118
HMS6	3.50	2.30	0.51	0.61	0.56	0.95	−0.097	−0.0230
HTG4	3.83	2.68	0.61	0.72	0.63	1.10	−0.140	0.0001
VHL20	6.00	4.48	0.80	0.84	0.77	1.60	−0.081	−0.0039
Mean	4.54	2.98	0.63	0.69	0.62	1.17	−0.116	−0.0098
SE	0.15	0.11	0.14	0.02	0.02	0.04	0.05	0.02

**Table 3:** Genetic diversity in Shagya Arabian subpopulations. Sample size (N), number of alleles, average number of alleles per population (Na), average effective number of alleles per population (Ne), observed heterozygosity (Ho), expected heterozygosity (He), allelic richness (AR), and inbreeding coefficient (*F*_IS_).

Breed	Acronym	N	Num. of Alleles	Na	Ne	Ho	He	AR	*F*_IS_
Dahoman	DAH	25	70	4.67	3.04	0.68	0.61	3.92	−0.120
Gazal	GAZ	24	67	4.47	2.97	0.70	0.63	3.84	−0.113
Ibrahim	IBR	37	77	5.13	3.09	0.69	0.64	3.91	−0.076
Kuhailan Zaid	KUH ZAID	21	67	4.46	2.87	0.65	0.59	3.76	−0.101
O`Bajan	O’BAJ	25	73	4.87	3.13	0.69	0.65	4.01	−0.066
Shagya	SHA	8	55	3.67	2.76	0.71	0.57	3.63	−0.233
Mean				4.54	2.98	0.69	0.62	3.84	−0.202
SE				0.15	0.11	0.02	0.02	0.13	0.22

**Table 4:** *F*-statistics of 15 microsatellite loci analyzed in the entire Shagya Arabian population in Bulgaria [42].

Locus	*F*_IS_	*F*_IT_	*F*_ST_
AHT4	−0.076	0.024	0.093
ASB2	−0.077	−0.032	0.042
HMS2	−0.048	0.029	0.073
HMS7	−0.183	−0.080	0.087
HTG6	−0.103	−0.033	0.064
AHT5	−0.158	−0.092	0.057
ASB23	−0.060	0.072	0.124
HMS3	−0.158	−0.028	0.112
HTG10	−0.213	−0.122	0.075
HTG7	−0.051	0.068	0.113
ASB17	−0.113	−0.061	0.047
HMS1	−0.139	−0.044	0.083
HMS6	−0.087	−0.044	0.040
HTG4	−0.148	−0.047	0.088
VHL20	−0.082	−0.021	0.057
Mean	−0.113	−0.027	0.077
SE	0.013	0.014	0.007

## Data Availability

The original contributions presented in this study are included in this article/the Supplementary Materials; further inquiries can be directed to the corresponding author.

## Supplementary Materials

The following supporting information can be downloaded at: https://www.mdpi.com/article/10.3390/vetsci12080776/s1, Table S1: Sample size (N), number of alleles (Na), number of effective alleles (Ne), Shannon’s information index (I), number of private alleles (Np) and fixation index (FST) over all loci for Shagya Arabian sire lines. Table S2: Hardy Weinberg (HW) equilibrium test in all studied microsatellite loci by Shagya Arabian horse lineages. Table S3: Pairwise Nei’s [39] genetic distances (above diagonal) and FST values (below diagonal) among six Shagya Arabian horse lineages. Table S4: Analysis of molecular variance (AMOVA) of Shagya Arabian horse line-ages based on genotyping of 15 the microsatellite markers.
