# Supplementary material for: Population Structure and Genetic Diversity Among Shagya Arabian Horse Genealogical Lineages in Bulgaria Based on Microsatellite Genotyping
Source: Vet Sci. 2025 Aug 19;12(8):776. doi: 10.3390/vetsci12080776 (PMC12390109; doi:10.3390/vetsci12080776)
Supplement: Supplementary file 1 [file vetsci-12-00776-s001.zip › vetsci-3747934-supplementary/Supplementary Table S1.pdf]

Supplementary Table S1. Sample size (N), number of alleles (Na), number of effective alleles (Ne), Shannon's information index (I), number of private alleles (Np) and fixation index (F_{ST}) over all loci for Shagya Arabian sire lines.

Sire lineage	Locus	N	Na	Ne	I	Np	F_{ST}
Dahoman	AHT4	25	5.000	2.998	1.274	0	-0.261
	ASB2	25	9.000	5.708	1.923	1	-0.067
	HMS2	25	5.000	3.109	1.319	0	-0.061
	HMS7	25	5.000	2.803	1.255	0	0.005
	HTG6	25	3.000	2.189	0.927	0	-0.105
	AHT5	25	4.000	1.778	0.805	0	-0.280
	ASB23	24	5.000	4.608	1.563	0	-0.064
	HMS3	25	5.000	3.102	1.295	0	-0.181
	HTG10	25	5.000	4.072	1.486	0	-0.060
	HTG7	25	2.000	1.220	0.325	0	-0.111
	ASB17	24	6.000	2.600	1.293	0	-0.083
	HMS1	25	4.000	2.000	0.897	0	-0.040
	HMS6	25	3.000	2.059	0.772	0	-0.166
	HTG4	25	4.000	2.615	1.127	0	-0.166
	VHL20	25	5.000	4.753	1.583	0	-0.216
Gazal	AHT4	24	5.000	2.924	1.299	0	-0.266
	ASB2	24	7.000	4.683	1.698	0	-0.060
	HMS2	24	5.000	1.973	0.987	0	0.070
	HMS7	24	4.000	2.612	1.074	0	-0.148
	HTG6	24	3.000	1.969	0.844	0	-0.101
	AHT5	24	4.000	2.499	1.053	0	-0.111
	ASB23	24	4.000	1.781	0.851	0	-0.141
	HMS3	24	6.000	2.873	1.366	0	-0.214
	HTG10	24	5.000	3.959	1.494	0	-0.115
	HTG7	24	2.000	1.917	0.671	0	0.216
	ASB17	24	4.000	3.657	1.334	0	-0.090
	HMS1	24	4.000	3.072	1.198	0	-0.112
	HMS6	24	3.000	2.577	1.020	0	-0.021
	HTG4	24	4.000	2.931	1.218	0	-0.265
	VHL20	24	7.000	5.189	1.742	0	-0.187
Ibrahim	AHT4	37	5.000	4.409	1.545	0	-0.049
	ASB2	37	8.000	5.599	1.830	0	-0.119
	HMS2	37	7.000	2.342	1.211	1	-0.038
	HMS7	37	6.000	3.492	1.427	0	-0.098
	HTG6	37	3.000	1.903	0.828	0	-0.082
	AHT5	37	5.000	2.837	1.154	1	-0.085
	ASB23	37	6.000	3.299	1.317	0	-0.008
	HMS3	37	5.000	2.421	1.144	0	-0.151
	HTG10	37	5.000	4.117	1.515	0	-0.142
	HTG7	37	3.000	1.930	0.826	1	-0.178

	ASB17	37	5.000	2.805	1.258	0	-0.092
	HMS1	37	5.000	2.318	0.982	1	-0.141
	HMS6	37	4.000	2.127	0.903	0	-0.071
	HTG4	37	4.000	2.620	1.055	0	0.082
	VHL20	37	6.000	4.187	1.540	0	0.006
	AHT4	21	6.000	4.046	1.529	0	-0.012
	ASB2	21	8.000	4.642	1.779	0	-0.214
	HMS2	21	4.000	1.418	0.618	0	0.031
	HMS7	21	4.000	3.920	1.376	0	-0.279
	HTG6	21	3.000	2.110	0.879	0	-0.086
	AHT5	21	2.000	1.747	0.619	0	-0.003
Kuhailan Zaid	ASB23	21	4.000	2.377	1.009	0	-0.068
	HMS3	21	6.000	2.739	1.292	0	-0.200
	HTG10	21	5.000	2.980	1.284	0	-0.290
	HTG7	21	2.000	1.324	0.410	0	-0.167
	ASB17	21	5.000	3.835	1.435	0	-0.224
	HMS1	21	4.000	3.000	1.209	0	0.071
	HMS6	21	3.000	1.956	0.792	0	-0.267
	HTG4	21	4.000	2.390	1.055	0	0.263
	VHL20	21	7.000	4.642	1.714	0	0.029
	AHT4	25	6.000	4.325	1.584	0	0.116
	ASB2	25	7.000	5.342	1.765	0	0.114
	HMS2	25	6.000	2.969	1.354	0	-0.146
	HMS7	25	5.000	2.345	1.078	0	-0.325
	HTG6	25	3.000	2.828	1.068	0	-0.052
	AHT5	25	4.000	2.495	1.123	0	-0.202
O`Bajan	ASB23	25	5.000	3.181	1.342	0	0.067
	HMS3	25	6.000	3.117	1.347	0	-0.178
	HTG10	25	5.000	3.453	1.356	0	-0.182
	HTG7	25	2.000	1.317	0.405	0	-0.163
	ASB17	25	6.000	3.360	1.395	0	-0.025
	HMS1	25	4.000	2.729	1.108	0	-0.136
	HMS6	25	4.000	2.822	1.197	0	0.071
	HTG4	25	4.000	2.822	1.151	0	-0.177
	VHL20	25	6.000	3.858	1.510	0	0.028
	AHT4	8	4.000	3.657	1.333	0	-0.032
	ASB2	8	6.000	4.571	1.630	0	-0.120
	HMS2	8	3.000	1.293	0.463	0	-0.103
	HMS7	8	4.000	3.282	1.282	0	-0.258
Shagya	HTG6	8	3.000	2.723	1.043	0	-0.185
	AHT5	8	3.000	2.462	0.974	0	-0.263
	ASB23	8	3.000	1.662	0.703	0	-0.255
	HMS3	8	4.000	2.612	1.157	0	-0.013
	HTG10	8	4.000	2.844	1.163	0	-0.542

HTG7	8	1.000	1.000	0.000	0	#N/A
ASB17	8	4.000	2.844	1.180	0	-0.157
HMS1	8	4.000	3.282	1.251	0	-0.438
HMS6	8	4.000	2.246	1.041	0	-0.127
HTG4	8	3.000	2.723	1.043	0	-0.580
VHL20	8	5.000	4.267	1.511	0	-0.143
Mean		4.544	2.980	1.175	0.05	-0.117
SE		0.15	0.11	0.04	0.10	0.014
