# Supplementary material for: Population Structure and Genetic Diversity Among Shagya Arabian Horse Genealogical Lineages in Bulgaria Based on Microsatellite Genotyping
Source: Vet Sci. 2025 Aug 19;12(8):776. doi: 10.3390/vetsci12080776 (PMC12390109; doi:10.3390/vetsci12080776)
Supplement: Supplementary file 1 [file vetsci-12-00776-s001.zip › vetsci-3747934-supplementary/Supplementary Table S2.pdf]

Supplementary Table S2. Hardy Weinberg (HW) equilibrium test in all studied microsatellite loci by Shagya Arabian horse lineages.

Locus/Lineage	DAH	GAZ	IBR	KUH ZAID	O'BAJ	SHA
AHT4	0.135	0.703	0.619	0.726	0.312	0.644
ASB2	0.720	0.939	0.610	0.025*	0.516	0.428
HMS2	0.376	0.598	0.606	0.034*	0.826	0.983
HMS7	0.826	0.553	0.888	0.296	0.756	0.480
HTG6	0.744	0.904	0.156	0.737	0.241	0.110
AHT5	0.706	0.684	0.795	0.990	0.623	0.198
ASB23	0.912	0.930	0.901	0.964	0.541	0.828
HMS3	0.547	0.729	0.894	0.893	0.902	0.776
HTG10	0.964	0.488	0.308	0.307	0.199	0.392
HTG7	0.579	0.290	0.546	0.445	0.416	0.00
ASB17	0.543	0.858	0.400	0.112	0.952	0.609
HMS1	0.912	0.747	0.932	0.464	0.513	0.238
HMS6	0.661	0.736	0.910	0.435	0.797	0.912
HTG4	0.241	0.440	0.203	0.156	0.510	0.141
VHL20	0.435	0.741	0.760	0.675	0.603	0.493

Significant at $^{*}p < 0.05$. Shagya Arabian horse lineages abbreviations could be seen in Table 4.
