# Supplementary material for: Population Structure and Genetic Diversity Among Shagya Arabian Horse Genealogical Lineages in Bulgaria Based on Microsatellite Genotyping
Source: Vet Sci. 2025 Aug 19;12(8):776. doi: 10.3390/vetsci12080776 (PMC12390109; doi:10.3390/vetsci12080776)
Supplement: Supplementary file 1 [file vetsci-12-00776-s001.zip › vetsci-3747934-supplementary/Supplementary Table S3.pdf]

Supplementary Table S3. Pairwise Nei's [39] genetic distances (above diagonal) and F_{ST} values (below diagonal) among six Shagya Arabian horse lineages.

DAH	GAZ	IBR	KUH ZAID	O'BAJ	SHA	
0.000	0.259	0.111	0.145	0.234	0.191	DAH
0.065	0.000	0.151	0.202	0.172	0.191	GAZ
0.030	0.037	0.000	0.097	0.148	0.151	IBR
0.040	0.056	0.028	0.000	0.179	0.170	KUH ZAID
0.054	0.044	0.036	0.046	0.000	0.187	O'BAJ
0.057	0.060	0.049	0.053	0.054	0.000	SHA

Abbreviations: Shagya Arabian horse lineages abbreviations could be seen in Table 4.
