# Supplementary material for: Population Structure and Genetic Diversity Among Shagya Arabian Horse Genealogical Lineages in Bulgaria Based on Microsatellite Genotyping
Source: Vet Sci. 2025 Aug 19;12(8):776. doi: 10.3390/vetsci12080776 (PMC12390109; doi:10.3390/vetsci12080776)
Supplement: Supplementary file 1 [file vetsci-12-00776-s001.zip › vetsci-3747934-supplementary/Supplementary Table S4.pdf]

Supplementary Table S4. Analysis of molecular variance (AMOVA) of Shagya Arabian horse lineages based on genotyping of 15 the microsatellite markers.

Source	d.f.	SS	MS	Est. Var.	% of Variation
Among populations	5	6771.532	1354.306	11.608	2%
Among individuals	134	110815.153	826.979	233.497	39%
Within individuals	140	50398.000	359.986	359.986	59%
Total	279	167984.686		605.090	100%

Abbreviations: d.f. - degrees of freedom; SS - sum of squares; MS - mean of squares; Est. Var. - estimated variance. F_{ST} = 0.019 ($p \geq 0.01$); F_{IS} = 0.393 ($p \geq 0.01$); F_{IT} = 0.405 ($p \geq 0.01$)
